# Supplementary material for: Potent Therapeutic Strategies for COVID-19 with Single-Domain Antibody Immunoliposomes Neutralizing SARS-CoV-2 and Lip/cGAMP Enhancing Protective Immunity
Source: Int J Mol Sci. 2023 Feb 17;24(4):4068. doi: 10.3390/ijms24044068 (PMC9966208; doi:10.3390/ijms24044068)
Supplement: Supplementary file 1 [file ijms-24-04068-s001.zip › ijms-2168259-supplementary.pdf]

Supporting Information

for

Potent Therapeutic Strategies for COVID-19 with Single-Domain Antibody Immunoliposomes Neutralizing SARS-CoV-2 and Lip/cGAMP Enhancing Protective Immunity

Yajun Zhou [1], Xing Lu [1], Xiaoqing Wang [1], Tianlei Ying[2] and Xiangshi Tan [1,*]

1 *Department of Chemistry, Fudan University, 2005 Songhu Road, Shanghai 200433, China*

2 *Institutes of Biomedical Sciences, School of Basic Medical Sciences, Fudan University, Shanghai 200032, China*

* *Correspondence: xstan@fudan.edu.cn*

(A)

MDHHHHHHSDSEVNQEAKPEVKPEVKPETHINLKVSDGSSEIF
FKIKKTTPLRRLMEAFAKRQGKEMDSLRFLYDGIRIQADQTPED
LDMEDNDIIEAHREQIGGATYQVQLQESGGGLVQAGGSLRLSCA
ASGRTFSEYAMGWFRQAPGKEREFVATISWSGGSTYYTDSVKG
RFTISRDNAKNTVYLQMNSLKPDDTAVYYCAAAGLGTVVSEWD
YDYYLDYWGQGTQVTVSS

(B)

Score	Expect	Method	Identities	Positives	Gaps
250 bits(639)	1e-83	Compositional matrix adjust.	125/127(98%)	125/127(98%)	2/127(1%)

```
Query  1    QVQLQESGGGLVQAGGSLRLSCAASGRTFSEYAMGWFRQAPGKEREFVATISWSGGSTYY  60
            QVQLQESGGGLVQAGGSLRLSCAASGRTFSEYAMGWFRQAPGKEREFVATISWSGGSTYY
Sbjct  1    QVQLQESGGGLVQAGGSLRLSCAASGRTFSEYAMGWFRQAPGKEREFVATISWSGGSTYY  60

Query  61   TDSVKGRFTISRDNAKNTVYLQMNSLKPDDTAVYYCAAAGLGTVVSEWDYDYYLDYWGQG  120
            TDSVKGRFTISRDNAKNTVYLQMNSLKPDDTAVYYCAAAGLGTVVSEWDYDY  DYWGQG
Sbjct  61   TDSVKGRFTISRDNAKNTVYLQMNSLKPDDTAVYYCAAAGLGTVVSEWDYDY--DYWGQG  118

Query  121  TQVTVSS  127
            TQVTVSS
Sbjct  119  TQVTVSS  125
```

Figure S1 (A)The sequence of Smt3-SARSVHH, red coded Smt3, blue coded SARSVHH. (B)The BLASTp of SARSVHH (Query sequence) with VHH-72 (Sbjct sequence).

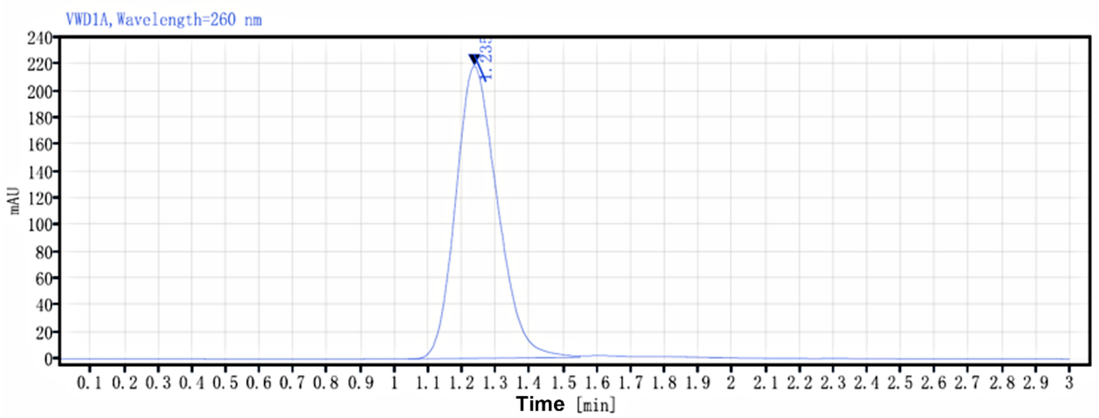

Signal: VWD1A, Wavelength=260 nm

Figure S2 A sample resulting chromatogram of the cGAMP by HPLC.

Table S1 Blood routine examination of normal Blood routine examination of normal and Lip/cGAMP addressed mice.

	**WBC (10^9/L)**	**lymph (10^9/L)**	**monocyte (10^9/L)**	**granulocyte (10^9/L)**	**RBC (10^{12}/L)**
Reference ranges	4.00~10.00	0.40~4.40	0.00~0.81	1.70~7.70	3.50~6.00
Normal	9.36 ± 2.33	7.35 ± 2.55	0.33 ± 0.16	3.87 ± 2.58	4.35 ± 1.88
Lip/cGAMP	11.32 ± 3.15	8.05 ± 2.75	0.36 ± 0.18	4.47 ± 2.39	5.88 ± 2.05

	**MCV**	**MCH**	**PLT**	**MPV**	**PDW**	**HCT**
Reference ranges	80.00~100.00	27.00~34.00	100.00~300.00	3.00~15.00	9.00~26.00	33.00~50.00
Normal	85.16 ± 8.70	15.35 ± 5.80	210.00 ± 20.00	6.35 ± 3.20	17.75 ± 3.80	46.80 ± 7.20
Lip/cGAMP	88.40 ± 7.60	15.55 ± 6.50	180.00 ± 35.00	6.40 ± 3.40	19.30 ± 4.10	48.40 ± 6.40

WBC： white blood cells; RBC： red blood cells; MCV： mean corpuscular volume; MCH： mean corpuscular hemoglobin; PLT： platelet; MPV： mean platelet volume; PDW： platelet distribution width; HCT： hematocrit。

Reference ranges was from *Laboratory animals and animal experimental technology*, edited by Mingsan Miao, China Press of Traditional Chinese Medicine, 1997.6.
